# Fast prediction of RNA-RNA interaction

**DOI:** 10.1186/1748-7188-5-5

**Published:** 2010-01-04

**Authors:** Raheleh Salari, Rolf Backofen, S Cenk Sahinalp

**Affiliations:** 1 School of Computing Science, Simon Fraser University, Burnaby, Canada; 2 Institute für Informatik, Albert-Ludwigs-Universität, Freiburg, Germany

## Abstract

**Background:**

Regulatory antisense RNAs are a class of ncRNAs that regulate gene expression by prohibiting the translation of an mRNA by establishing stable interactions with a target sequence. There is great demand for efficient computational methods to predict the specific interaction between an ncRNA and its target mRNA(s). There are a number of algorithms in the literature which can predict a variety of such interactions - unfortunately at a very high computational cost. Although some existing target prediction approaches are much faster, they are specialized for interactions with a single binding site.

**Methods:**

In this paper we present a novel algorithm to accurately predict the minimum free energy structure of RNA-RNA interaction under the most general type of interactions studied in the literature. Moreover, we introduce a fast heuristic method to predict the specific (multiple) binding sites of two interacting RNAs.

**Results:**

We verify the performance of our algorithms for joint structure and binding site prediction on a set of known interacting RNA pairs. Experimental results show our algorithms are highly accurate and outperform all competitive approaches.

## Background

Regulatory non-coding RNAs (ncRNAs) play an important role in gene regulation. Studies on both prokaryotic and eukaryotic cells show that such ncRNAs usually bind to their target mRNA to regulate the translation of corresponding genes. Many regulatory RNAs such as microRNAs and small interfering RNAs (miRNAs/siRNAs) are very short and have full sequence complementarity to the targets. However some of the regulatory antisense RNAs are relatively long and are not fully complementary to their target sequences. They exhibit their regulatory functions by establishing stable joint structures with target mRNA initiated by one or more loop-loop interactions.

In this paper we present an efficient method for the RNA-RNA interaction prediction (RIP) problem with multiple binding domains. Alkan et al. [1] proved that RIP, in its general form, is an NP-complete problem and provided algorithms for predicting specific types of interactions and two relatively simple energy models - under which RIP is polynomial time solvable. We focus on the same type of interactions, which to the best of our knowledge, are the most general type of interactions considered in the literature; however the energy model we use is the joint structure energy model recently presented by Chitsaz et al. [2] which is more general than the one used by Alkan et al.

In what follows below, we first describe a combinatorial algorithm to compute the minimum free energy joint structure formed by two interacting RNAs. This algorithm has a running time of *O*(*n*^6^) and uses *O*(*n*^4^) space - which makes it impractical for long RNA molecules. Then we present a fast heuristic algorithm to predict the joint structure formed by interacting RNA pairs. This method provides a significant speedup over our combinatorial method, which it achieves by exploiting the observation that the independent secondary structure of an RNA molecule is mostly preserved even after it forms a joint structure with another RNA. In fact there is strong evidence [3,4] suggesting that the probability of an ncRNA binding to an mRNA target is proportional to the probability of the binding site having an unpaired conformation. The above observation has been used by different methods for target prediction in the literature (see below for an overview). However, most of these methods focus on predicting interactions involving only a single binding site, and are not able to predict interactions involving multiple binding sites. In contrast, our heuristic approach can predict interactions involving multiple binding sites by: (1) identifying the collection of accessible regions for both input RNA sequences, (2) using a matching algorithm, computing a set of "non-conflicting" interactions between the accessible regions which have the highest overall probability of occurrence.

Note that an accessible region is a subsequence in an RNA sequence which, with "high" probability, remain unpaired in its secondary structure. Our method considers the possibility of interactions being formed between one such accessible region from an RNA sequence with more than one such region from the other RNA sequence. Thus, in step (1), it extends the algorithm by Mückstein et al. for computing the probability of a specific region being unpaired [5] to compute the joint probability of two (or more) regions remaining unpaired. Because an accessible region from an RNA typically interacts with no more than two accessible regions from the other RNA, we focus on calculating the probability of at most two regions remaining unpaired: within a given an RNA sequence of length *n*, our method can calculate the probability of any pair of regions of length ≤ *w *each, in *O*(*n*^4^.*w*) time and *O*(*n*^2^) space. In step (2), on two input RNA sequences of length *n *and *m *(*n *≤ *m*), our method computes the most probable non-conflicting matching of accessible regions in *O*(*n*^2^.*w*^4 ^+ *n*^3^/*w*^3^) time and *O*(*w*^4 ^+ *n*^2^/*w*^2^) space.

### Related work

Early attempts to compute the joint structure of interacting RNAs started by concatenating the two interacting RNA sequences and treated them as a single sequence PairFold[6] and RNAcofold[7]. Dirks et al. present a method, as a part of NUPack, that concatenates the input sequences in some order, carefully considering symmetry and sequence multiplicities, and computes the partition function for the whole ensemble of complex species [8]. As these methods typically use secondary structure prediction methods that do not allow pseudoknots, they fail to predict joint structures formed by non-trivial interactions between a pair of RNAs.

Another set of methods ignore internal base-pairing in both RNAs, and compute the minimum free energy secondary structure for their hybridization (RNAhybrid[9], UNAFold[10,11], and RNAduplex from Vienna package [7]). These approaches work only for simple cases involving typically very short strands.

A further set of studies aim to compute the minimum free energy joint structure between two interacting RNAs. For example Pervouchine [12] devised a dynamic programming algorithm to maximize the number of base pairs among interacting strands. A follow up work by Kato et al. [13] proposed a grammar based approach to RNA-RNA interaction prediction. More generally Alkan et al. [1] studied the joint secondary structure prediction problem under three different models: 1) base pair counting, 2) stacked pair energy model, and 3) loop energy model. Alkan et al. proved that the general RNA-RNA interaction prediction under all three energy models is an NP-hard problem. Therefore, they suggested some natural constraints on the topology of possible joint secondary structures which are satisfied by all examples of complex RNA-RNA interactions in the literature. The resulting algorithms compute the optimum structure among all possible joint secondary structures that do not contain pseudoknots, crossing interactions, and *zigzags *(please see [1] for the exact definition). In fact the last set of algorithms above are the only methods that have the capability to predict joint secondary structures with multiple loop-loop interactions. However, these algorithms all requires significant computational resources (*O*(*n*^6^) time and *O*(*n*^4^) spaces) and thus are impractical for sequences of even modest length.

A final group of methods are based on the observation that interaction is a multi step process [14] that involves: 1) unfolding of the two RNA structures to expose the bases needed for hybridization, 2) the hybridization at the binding site, and 3) restructuring of the complex to a new minimum free energy conformation. The main aim of these methods is to identify the potential binding sites which are going to be unfolded in order to form interactions. One such method presented by Alkan et al. [1], extends existing loop regions in independent structures to find potential binding sites. RNAup[15] presents an extension of the standard partition function approach to compute the probabilities that a sequence interval remains unpaired. IntaRNA[16] considers not only accessibility of a binding sites but also the existence of a seed to predict potential binding sites. All of these methods achieve reasonably high accuracy in predicting interactions involving single binding sites; however, their accuracy levels are not very high when dealing with interactions involving multiple binding sites.

## Methods

We address the RNA-RNA Interaction Problem (RIP) based on the interaction energy model proposed by Chitsaz et al. [2] over the type of interaction considered by Alkan et al. [1]. Our algorithm computes the minimum free energy joint secondary structure that does not contain pseudoknots, crossing interactions, and *zigzags*. The zigzag constraint simply states that if two substructures from two RNAs interact, then one substructure must subsume the other.

### RNA-RNA joint structure prediction

Recently Chitsaz et al. [2] present an energy model for joint structure of two nucleic acid strands over the type of interaction introduced by Alkan et al. [1]. Based on the presented energy model they propose an algorithm that consider all possible joint secondary structures to compute the partition function for two interacting nucleic acid strands. The specified algorithm with some minor changes can be used to compute the minimum free energy joint structure of two interacting nucleic acid strands. Following we shortly describe the dynamic programming algorithm to predict the minimum free energy RNA-RNA interaction. We are given two RNA sequences **R **and **S **of lengths *n *and *m*. Strand **R **is indexed from 1 to *n *in 5' to 3' direction and **S **is indexed from 1 to *m *in 3' to 5' direction. Note that the two strands interact in opposite directions, i.e. **R **in 5' → 3' with **S **in 3' ← 5' direction. Each nucleotide is paired with at most one nucleotide in the same or the other strand. We refer to the *i*^*th*^nucleotide in **R **and **S **by *i*_*R *_and *i*_*S *_respectively. The subsequence from the *i*^*th *^nucleotide to the *j*^*th*^nucleotide in one strand is denoted by [*i*, *j*]. We denote a base pair between the nucleotides *i *and *j *by *i*·*j*. *MFE*(*i, j*) denotes the minimum free energy structure of [*i*, *j*], and *MFE*(*i*_*R*_, *j*_*R*_, *i*_*S*_, *j*_*S*_) denotes the minimum free energy joint structure of [*i*_*R*_, *j*_*R*_] and [*i*_*S*_, *j*_*S*_].

**Figure 1:**
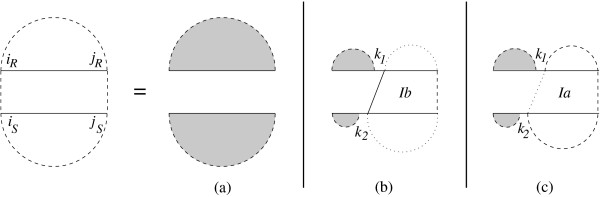
**Recursion for joint secondary structure of subsequences [*i*_*R*_, *j*_*R*_] and [*i*_*S*_, *j*_*S*_]**. Case *a *constitutes no interaction. In case *b*, the leftmost interaction bond is not closed by any base pair. In case *c*, the leftmost interaction bond is covered by base pair in at least one subsequence.

Figure 1 shows the recursion diagram of the *MFE *joint structure of [*i*_*R*_, *j*_*R*_] and [*i*_*S*_, *j*_*S*_]. In this figure a horizontal line indicates the phosphate backbone, a dashed curved line encloses a subsequence and denotes its two terminal bases which may be paired or unpaired. A solid vertical line indicates an interaction base pair, a dashed vertical line denotes two terminal bases which may be base paired or unpaired, and a dotted vertical line denotes two terminal bases which are assumed to be unpaired. Grey regions indicate a reference to the substructure of single sequences.

The joint structure of two subsequences derived from one of the following cases. The first possibility is when there is no interaction between the two subsequences. If there are some interaction bonds, the structure has two cases: either the leftmost bond is closed by base pair in at least one of the subsequences or not. If the joint structure starts with a bond which is not closed by any base pair we denote the case by *Ib*, otherwise the structure starts with a bond which is closed by base pair in at least one subsequence and the case is denoted by *Ia*. Therefore, *MFE*(*i*_*R*_, *j*_*R*_, *i*_*S*_, *j*_*S*_) is calculated by the following dynamic programming:(1)

in which *MFE*^*Ib*^(*k*_1_, *j*_*R*_, *k*_2_, *j*_*S*_) is the minimum free energy for the joint structure of [*k*_1_, *j*_*R*_] and [*k*_2_, *j*_*S*_] assuming *k*_1_·*k*_2 _is an interaction bond, and *MFE*^*Ia*^(*k*_1_, *j*_*R*_, *k*_2_, *j*_*S*_) is the minimum free energy for the joint structure of [*k*_1_, *j*_*R*_] and [*k*_2_, *j*_*S*_] assuming the leftmost interaction bond is covered by a base pair in at least one subsequence. The corresponding dynamic programing for computing the *MFE*^*Ib *^and *MFE*^*Ia *^can be derived from the cases explained in [2] in a similar way.

Similar to the partition function algorithm, the minimum free energy joint structure prediction algorithm has *O*(*n*^6^) running time and *O*(*n*^4^) space requirements. However the algorithm is highly accurate (see experimental results), but it requires substantial computational resources. Thus it could be prohibitive for predicting the joint secondary structures of long RNA molecules. In next section we present a fast heuristic algorithm to predict RNA-RNA interaction without applying any restriction on type of interaction and energy model.

### RNA-RNA binding sites prediction

Our heuristic algorithm for prediction of RNA-RNA interactions involving multiple binding sites is based on the idea that the external interactions mostly occur between unpaired regions of two RNA structures. The heuristic algorithm contains the following steps:

• Predict highly accessible regions in each strands. These regions include the loop regions in native structure of RNA strand. In order to predict accessible regions we chose all the regions which remain unpaired with high probability.

• Predict the optimal non-conflicting interactions between the accessible regions. For every pair of accessible regions of two interacting RNAs a cost of interaction is calculated. Then a matching algorithm runs to find the minimum cost non-conflicting subset of interactions.

#### Accessible regions

For a single RNA sequence an accessible region is a subsequence that remains unpaired in equilibrium with high probability. The probability of an unpaired region can be calculated based on the algorithm presented in RNAup [5]. Since we are interested in multiple unpaired regions, we need to consider the joint probabilities for all possible subsets of intervals. However, computation of all joint probabilities requires substantial time and space and thus in this paper we only consider the joint probability of two unpaired subsequences as well as the probability of an unpaired subsequence.

**Figure 2:**
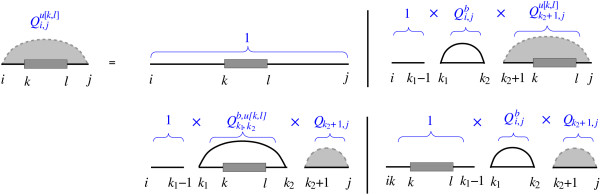
**Recursion for partition function of subsequence [*i*, *j*] while [*k*, *l*] remains unpaired**. Either the subsequence [*i*, *j*] is empty with recursion energy *G *= 0, or there exists one or more pairs with leftmost base pair *k*1·*k*2. There are three possibilities for the position of base pair *k*1·*k*2 and unpaired interval [*k*, *l*].

Denoting the set of secondary structures in which the sequence interval [*k*, *l*] remains unpaired by *S*^*u *[*k*, *l*]^, the corresponding partition function is(2)

where *R *is the universal gas constant and *T *is the temperature. In order to compute the *Q*^*u *[*k*, *l*]^, the standard recursion for the partition function folding algorithm [17] can be extended based on the recursion cases in Figure 2. Therefore,(3)

where *i *≤ *k *≤ *l *≤ *j *and *k*_1_·*k*_2 _is the leftmost base pair. Note that without loss of generality we assumed *i *≤ *k *≤ *l *≤ *j*. Clearly if [*k*, *l*] is not a subsequence of [*i*, *j*], we have . In fact  for any arbitrary interval [*k*, *l*] is equivalent to  such that [*k'*, *l'*] is the common subsequence between [*i*, *j*] and [*k*, *l*].

Partition functions  (where *i*·*j *is a base pair) and  (where [*i*, *j*] is inside a multiloop and constitutes at least one base pair) while the interval [*k*, *l*] remains unpaired are derived from the standard algorithm in a similar way. Furthermore, probability of a base pair *p*·*q *while [*k*, *l*] remains unpaired, ℙ(*p*·*q*|*u *[*k*, *l*]), can be calculated by applying the McCaskill algorithm [17] for computing the base pair probability on *Q*^*u *[*k*, *l*]^. It is easy to see that the desired partition function *Q*^*u *[*k*, *l*] ^and base pair probability ℙ(*p*·*q*|*u *[*k*, *l*]) are computed in same time and space complexity as the standard algorithm by McCaskill - it has *O*(*n*^3^) time and *O*(*n*^2^) space complexity.

Mückstein et al. [5] introduce an algorithm to compute the probability of unpaired region ℙ(*u *[*i*, *j*]) for a given sequence interval [*i*, *j*]. Here, we extend the specified algorithm to compute ℙ(*u *[*i*, *j*]|*u *[*k*, *l*]) which is the probability of unpaired sequence interval [*i*, *j*] while interval [*k*, *l*] remains unpaired. Clearly if some part of [*i*, *j*] is within the interval [*k*, *l*], the corresponding probability for that part is equal to one. Hence, for computing the probability only those parts of [*i*, *j*] which are exterior to [*k*, *l*] should be considered. Here, without loss of generality we assume *k *≤ *l *≤ *i *≤ *j*.

**Figure 3:**
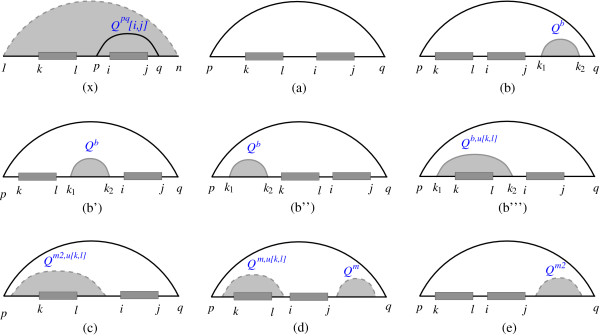
**Cases of unpaired interval [*i*, *j*] within a loop enclosed by *p*·*q *while [*k*, *l*] remains unpaired**. In case (x), interval [*k*, *l*] is outside of substructure [*p*, *q*], but its effect on the probability of base pair *p*·*q *should be considered. For the other cases substructure [*p*, *q*] contains interval [*k*, *l*]. Base pair *p*·*q *can close different loop types (a) hairpin, (b-b"') internal loop, and (c-e) multiloop. Cases (b-b"') refer to the four possibilities for the position of interior base pair *k*1·*k*2 and unpaired intervals [*k*, *l*] and [*i*, *j*]. If base pair *p*·*q *closes a multiloop, unpaired intervals [*k*, *l*] and [*i*, *j*] can have three different conformations (c-e).

For an unpaired interval [*i*, *j*] there are two general cases: either it is not closed by any base pair, or it is part of a loop. Figure 3 summarizes the cases of unpaired interval [*i*, *j*] as a part of the loop enclosed by base pair *p*·*q *while interval [*k*, *l*] remains unpaired. In case *x *interval [*p*, *q*] does not contain interval [*k*, *l*], and in the other cases (*a *- *e*) interval [*k*, *l*] lies in interval [*p*, *q*]. Probability ℙ(*u *[*i*, *j*]|*u *[*k*, *l*]) can be calculated as follows:(4)

The partition function *Q*^*pq *^[*i*, *j*] which is introduced by Mückstein et al. considers all structures on [*p*, *q*] while [*i*, *j*] is part of the loop closed by base pair *p*·*q*. The quantity *Q*^*pq*, *u *[*k*, *l*] ^[*i*, *j*] is a variant of *Q*^*pq *^[*i*, *j*] while [*k*, *l*] lies in [*p*, *q*]. Recursion of *Q*^*pq*, *u *[*k*, *l*] ^[*i*, *j*] on cases (*a *- *e*) displayed in Figure 3, is based on different types of loop and position of [*k*, *l*]. Therefore, we have(5)

where *Q*^*m*2 ^is the partition function of a subsequence inside a multiloop that constitutes at least two base pairs. *Q*^*m*2 ^which is introduced in Mückstein et al. algorithm can be extended to calculate *Q*^*m*2, *u *[*k*, *l*]^:(6)

where  is the partition function of a subsequence inside a multiloop that constitutes exactly one base pair such that *k*_1 _is one terminal of that base pair. Recursion of  can be simply derived from. recursion of . Therefore, the joint probability of two unpaired regions is obtained using(7)

The Mückstein et al. algorithm requires *O*(*n*^3^) running time and *O*(*n*^2^) space complexity to compute the probability of unpaired region ℙ(*u *[*i*, *j*]) for every possible interval [*i*, *j*] assuming the interval length is limited to size *w*. Using the extended algorithm, given sequence interval [*k*, *l*] computing ℙ(*u *[*i*, *j*], *u *[*k*, *l*]) for every possible interval [*i*, *j*] requires the same time and space complexity. Note that for each interval [*k*, *l*], *Q*^*u *[*k*, *l*] ^should be computed separately. Since there are *O*(*n.w*) different intervals for a limited interval length *w*, with *O*(*n*^4^.*w*) running time and *O*(*n*^2^) space complexity we are able to compute the joint probabilities for all pairs of unpaired regions. The same idea can be used to compute the joint probability of multiple unpaired regions. However, considering each extra interval increases the running time by a factor of *O*(*n.w*).

All the regions that have probability of being unpaired more than some fixed threshold are selected as accessible regions *r*_*i *_from sequecen **R **(as well as *s*_*j *_from sequecen **S**). For two consecutive intervals, *r*_*i *_= [*k*_*i*_, *l*_*i*_] and *r*_*i*_+1 = [*k*_*i*+1_, *l*_*i*+1_], in order to decide whether the concatenated region should be considered the joint probability ℙ(*u *[*r*_*i*_], *u *[*r*_*i*+1_]) and single probability ℙ(*u *[*k*_*i*_...l_*i*+1_]) are compared. The selected intervals are extended by some limited number of nucleotides (< 5) in each side.

#### Interaction matching algorithm

Given two lists of non-overlapping accessible regions *T*_*R *_= {*r*_1_, *r*_2_, ..., *r*_*n'*_} and *T*_*S *_= {*s*_1_, *s*_2_, ..., *s*_*m'*_}sorted according to their orders in interacting sequences **R **and **S**, we aim to calculate the optimal set of interactions between the accessible regions under the following constraints:

• Each accessible region can interact with at most two accessible regions from the other sequence.

• There is no crossing interaction.

For computing the interaction between accessible regions, IntaRNA minimizes the free energy of interaction and RNAup maximizes the probability of interaction while no internal base pair is allowed. Both approaches use RNAhybrid energy model for interaction. As mentioned before, we select a set of high probable unpaired intervals and extend them by some limited number of nucleotides. This extension is motivated by the observation that suggests usually the hybridization initiated at the accessible regions, and then some adjacent internal base pairs open up to form new interactions and make the complex more stable [14]. In order to not always prefer interaction rather than internal base pair in accessible regions, our method allows internal base pairs as well as interactions between accessible regions. We consider both options of minimizing the free energy of interaction and maximizing the probability of interaction while the interaction energy model introduced by [2] has been used.

Let  be the partition function over all possible joint structures of two subsequences *r*_*i *_and *s*_*j*_, which can be calculated by interaction between accessible piRNA[2]. Define  as the partition function for the set of joint structures that contain some interactions. We denote two interacting subsequences *r*_*i *_and *s*_*j *_by *r*_*i *_∘ *s*_*j*_. Therefore, probability of interaction for two accessible regions *ri *and *s*_*j *_is considered as . The interaction between two accessible regions *r*_*i *_and *s*_*j *_is considered if and only if ℙ(*r*_*i *_∘ *s*_*j*_) > 1/2, i.e. the probability of interaction for two accessible regions is higher than the probability of forming independent single structures. In this case the ensemble free energy of interacting joint structure for the two accessible regions is

Also the minimum free energy of interaction for two accessible regions *r*_*i *_and *s*_*j*_, *MFE*(*r*_*i*_, *s*_*j*_), can be calculated by using the dynamic programming algorithm explained in previous section. If our goal is to minimize the free energy of interaction, accessible regions *r*_*i *_and *s*_*j *_are considered to be able to interact if and only if *MFE*(*r*_*i*_, *s*_*j*_) <*MFE*(*r*_*i*_) + *MFE*(*s*_*j*_), i.e. there are some interaction bonds in the minimum free energy joint structure.

Let *E*_*u*_(*ri*) as the energy difference between the complete ensemble and the ensemble in which the interacting subsequences are left unpaired for accessible region *r*_*i*_. We have

The cost of interaction between two accessible regions *r*_*i *_and *s*_*j*_, *C*(*r*_*i*_, *s*_*j*_), is the sum of the following terms: (i) *E*_*u*_(*r*_*i*_), (ii) *E*_*u*_(*s*_*j*_), and (iii) *E*_*I*_(*r*_*i*_, *s*_*j*_) or *MFE*(*r*_*i*_, *s*_*j*_). Cost of interaction between an accessible region *r*_*i *_and two other accessible regions *s*_*k *_and *s*_*j*_is defined as

where *s*_*k*_*s*_*j *_is the concatenation of two subsequences, and *E*_*u*_(*s*_*k*_, *s*_*j*_) = (*-RT*) ln(ℙ(*u *[*s*_*k*_], *u *[*s*_*j*_])). Similarly the cost of interaction between two accessible regions from **R **and one accessible region from **S **is defined. Also the cost of interaction where minimum free energy *MFE*(*r*_*i*_, *s*_*k*_*s*_*j*_) is used instead of ensemble energy *E*_*I*_(*r*_*i*_, *s*_*k*_*s*_*j*_) can be defined in a similar way.

With *H*(*i*, *j*), we denote the minimum cost non-conflicting set of interactions between the accessible regions {*r*_1_, ..., *r*_*i*_} and {*s*_1_, ..., *s*_*j*_}. The following dynamic programming computes *H*(*i*, *j*):(8)

where 1 ≤ *i *≤ *n' *and 1 ≤ *j *≤ *m'*. The algorithm starts by calculating *H*(1, 1) and explores all *H*(*i*, *j*) by increasing *i *and *j *until *i *= *n' *and *j *= *m'*. The DP algorithm has *O*(*n'*^2^.*m' *+ *n'.m'*^2^) time and *O*(*n'.m'*) space requirements. Also we need *O*(*n'.m'.w*^6^) time and *O*(*w*^4^) space to compute the cost of interaction for every pair of accessible regions. Assuming *n' *≥ *m' *and *n' *≤ *n*/*w*, we can conclude that this step of the algorithm requires *O*(*n*^2^.*w*^4 ^+ *n*^3^/*w*^3^) time and *O*(*w*^4 ^+ *n*^2^/*w*^2^) space.

**Figure 4:**
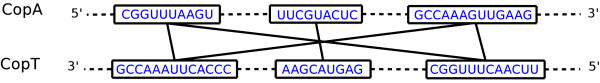
**An example for interaction matching algorithm**. Possible interactive accessible regions of CopA and CopT.

**Figure 5:**
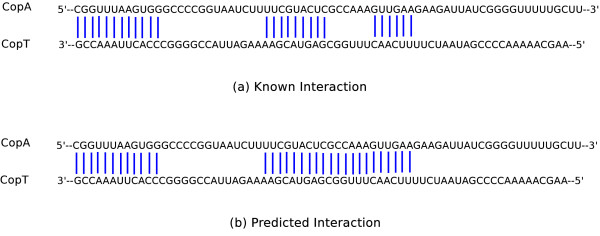
**Interaction between CopA and CopT**. (a) Known interaction bonds. (b) Predicted interaction bonds. Here, all internal base pairs are ignored and only the interaction bonds are displayed.

CopA-CopT is a well known antisense RNA-target complex observed in *E. coli *[18]. The joint structure of CopA-CopT contains two disjoint binding sites. Figure 4 shows the identified accessible regions in CopA and CopT. Two regions connected by an edge are able to interact. Figure 5 shows the known and predicted interaction bonds between CopA and CopT. Note that internal bonds of both RNAs are not displayed in this figure.

## Results and Discussion

### Dataset

In our experiments we use a dataset of 23 known RNA-RNA interactions which contains two recently compiled test sets. The first set includes 5 pairs of RNAs which are known to have loop-loop interactions and have been used by Kato et al. [13] to evaluate the proposed grammatical parsing approach for RNA-RNA joint structure prediction. The next 18 sRNA-target pairs are compiled and used as test set by Busch et al. in IntaRNA[16]. In our dataset OxyS-fhlA and CopA-CopT are the only ones that have two disjoint binding sites.

### Joint secondary structure prediction

In our first experiment, we assess the performance of our prediction algorithm for minimum free energy joint structure. For this purpose we use the 5 RNA-RNA complexes from Kato et al. [13] test set. We compare our results with two other state-of-the-art methods for joint structure prediction: (1) the grammatical approach by Kato et al. [13] (denoted by EBM as energy-based model), and (2) the DP algorithms for two energy models presented by Alkan et al. [1] (denoted by SPM as stacked-pair model and LM as loop model).

In order to estimate the accuracy of prediction, we measure the sensitivity and PPV defined as follows:(9)

As another measure of accuracy we calculate F-measure which considers both sensitivity and PPV. F-measure is the harmonic mean of sensitivity and PPV, and its formula is as follows:(11)

Table 1 shows the accuracy results of our method and the other competitors for joint structure prediction. We refer to our method by inRNAs as an algorithm for prediction the interactions between RNAs. As it can be seen in Table 1, our method based on the three accuracy measures outperforms the competitors. For Tar-Tar* and R1inv-R2inv pairs that both RNAs are relatively short (~20 nt), all methods are accurate enough. However, for DIS-DIS which is not still long (35 nt), only our method is able to predict the interaction while the other approaches return no interaction. CopA-CopT and IncRNA_54_-RepZ are a bit longer (~60 nt); CopA-CopT has two disjoint binding sites and IncRNA_54_-RepZ has a continuous binding site. Our method outperforms the others in predicting the joint structure of CopA-CopT, while IncRNA_54_-RepZ is predicted more accurately by EBM. We do not compare the running time between these methods due to the fact that each one uses different platform and hardware. Our method on one Sun Fire processor X4600 2.6 GHz with 64 GB RAM runs for ~4000(*sec*) to predict the joint structures of CopA-CopT and IncRNA_54_-RepZ.

**Table 1:** Prediction accuracy of competitive RNA-RNA joint secondary structure prediction methods.

	Sensitivity				PPV				F-measure			
	
RNA-RNA interaction pairs	**inRNAs**	EBM	SPM	LM	**inRNAs**	EBM	SPM	LM	**inRNAs**	EBM	SPM	LM
CopA-CopT	1.000	0.909	0.955	0.864	0.846	0.800	0.778	0.760	0.917	0.851	0.857	0.809
DIS-DIS	1.000	0.786	0.786	0.786	1.000	0.786	0.786	0.786	1.000	0.786	0.786	0.786
IncRNA_54_-RepZ	0.875	0.917	0.875	0.875	0.792	0.830	0.778	0.778	0.831	0.871	0.824	0.824
R1inv-R2inv	0.900	0.900	1.000	1.000	0.900	0.947	1.000	1.000	0.900	0.923	1.000	1.000
Tar-Tar*	1.000	1.000	1.000	1.000	0.875	0.933	0.875	0.875	0.933	0.965	0.933	0.933

Average	0.955	0.902	0.923	0.905	0.883	0.859	0.843	0.840	0.916	0.879	0.880	0.870

This Table shows the sensitivity, PPV and F-measure for RNA-RNA joint secondary structure prediction by (1) inRNAs, (2) the grammatical approach by Kato et al. [13] (denoted by EBM as energy-based model), and (3) the DP methods for two models presented by Alkan et al. [1] (denoted by SPM as stacked-pair model and LM as loop model). The dataset is compiled by Kato et al. [13].

### Binding sites prediction

In another experiment, we test the performance of our heuristic algorithm for interaction prediction. In order to identify the set of accessible regions in each sequence we set *w *= 25 and use *E*_*u *_< min{*E*_*u*_} + 2(*kcal/mol*) as cutoff. For assessing the predictive power of our algorithm, we compare our algorithm with IntaRNA[16] and RNAup[15]. Based on the experimental results presented by IntaRNA, both IntaRNA and RNAup which incorporate accessibility of target regions, perform better than the other competitive programs (TargetRNA[19], RNAhybrid[9], and RNAplex[20]).

The results of these two programs for the first 18 RNA pairs are as presented in [16]. For the next 5 RNA pairs, we run IntaRNA with its default settings and RNAup with the same setting that has been used by the experiment in [16] - RNAup has been run using parameter -b which considers the probability of unpaired regions in both RNAs and the maximal length of interaction to 80. In order to estimate accuracy of the programs, we measure the sensitivity, PPV and F-measure such that only interacting base pairs are considered.

Table 2 shows the results of our programs as well as IntaRNA and RNAup. In this dataset OxyS-fhlA and CopA-CopT are the only ones that have two disjoint binding sites, and our method clearly outperforms IntaRNA and RNAup by up to 30% improvement in F-measure. For the OxyS-fhlA complex with two loop-loop interactions, our method is able to find both binding sites. However, the other methods find only one of the binding sites. For CopA-CopT complex which contains one loop-loop interaction and one uncovered interaction site, again our method finds both binding sites. IntaRNA predicts one continues long binding site and RNAup predicted only the binding site within the loop-loop interaction. Another interesting case is GcvB-gltI complex. Both RNAup and IntaRNA can not predict any correct bond for GcvB-gltI, since they missed the binding site. However, IntaRNA can get 80% accuracy by considering the first suboptimal prediction which is close to the accuracy that we have achieved. In overall, the results demonstrate that our method predicts RNA-RNA interactions more accurately in compare to the competitive methods.

**Table 2:** Prediction accuracy of competitive RNA-RNA binding sites prediction methods.

	Sensitivity			PPV			F-measure		
	
RNA-RNA interaction pairs	**inRNAs**	**IntaRNA**	**RNAup**	**inRNAs**	**IntaRNA**	**RNAup**	**inRNAs**	**IntaRNA**	**RNAup**
CopA-CopT	0.889	1.000	0.556	0.828	0.391	0.652	0.857	0.562	0.600
DIS-DIS	1.000	1.000	1.000	1.000	1.000	1.000	1.000	1.000	1.000
IncRNA_54_-RepZ	1.000	0.738	0.750	0.889	0.850	0.857	0.941	0.790	0.800
R1inv-R2inv	1.000	1.000	1.000	0.778	1.000	0.778	0.875	1.000	0.875
Tar-Tar*	1.000	1.000	1.000	0.833	0.833	0.833	0.909	0.909	0.909
DsrA-RpoS	0.808	0.808	0.808	0.778	0.778	0.778	0.793	0.793	0.793
GcvB-argT	0.950	0.950	0.900	0.864	0.950	0.947	0.905	0.950	0.923
GcvB-dppA	1.000	1.000	1.000	0.850	0.586	0.459	0.919	0.739	0.629
GcvB-gltI	0.750	0.000	0.000	0.500	0.000	0.000	0.600	0.000	0.000
GcvB-livJ	0.634	0.955	0.955	0.824	0.955	0.955	0.717	0.955	0.955
GcvB-livK	0.540	0.542	0.542	0.570	0.565	0.565	0.555	0.553	0.553
GcvB-oppA	1.000	1.000	1.000	0.733	0.957	0.957	0.846	0.978	0.978
GcvB-STM4351	0.760	0.760	0.880	1.000	0.905	0.957	0.864	0.826	0.917
IstR-tisAB	0.722	0.879	0.667	1.000	0.960	1.000	0.839	0.918	0.800
MicA-ompA	1.000	1.000	1.000	1.000	1.000	1.000	1.000	1.000	1.000
MicA-lamB	1.000	1.000	0.826	1.000	0.821	0.704	1.000	0.902	0.760
MicC-ompC	1.000	1.000	0.727	1.000	0.537	0.410	1.000	0.699	0.524
MicF-ompF	0.960	0.960	0.800	0.960	0.960	0.952	0.960	0.960	0.869
OxyS-fhlA	0.813	0.500	0.375	1.000	1.000	1.000	0.897	0.667	0.545
RyhB-sdhD	0.618	0.588	0.794	0.955	1.000	0.794	0.750	0.741	0.794
RyhB-sodB	1.000	1.000	1.000	1.000	0.818	0.900	1.000	0.900	0.947
SgrS-ptsG	0.566	0.739	0.739	0.765	1.000	1.000	0.651	0.850	0.850
Spot42-galK	0.432	0.409	0.523	0.760	0.643	0.523	0.551	0.500	0.523

Average	0.845	0.819	0.776	0.865	0.805	0.784	0.845	0.791	0.763

This Table shows the sensitivity, PPV and F-measure for RNA-RNA binding sites prediction by (1) inRNAs, (2) IntaRNA[16], and (3) RNAup [15]. The dataset is compiled by Kato et al. [13] and Busch et al. [16].

## Conclusions

This paper introduce a fast algorithm for RNA-RNA interaction prediction. Our heuristic algorithm for the RNA-RNA interaction prediction problem incorporates the accessibility of multiple unpaired regions, and a matching algorithm to compute the optimal set of interactions involving multiple binding sites. The algorithm requires *O*(*n*^4^.*w*) running time and *O*(*n*^2^) space complexity. Note that the simplified version that allows each accessible region interact with at most one accessible region from the other sequence can be done in *O*(*n*^3^) running time. The main advantage of our method is its ability to predict multiple binding sites which have been predictable only by expensive algorithms [1,13] so far. On a set of several known RNA-RNA complexes, our proposed algorithm shows a reliable accuracy. Especially, for complexes with multiple binding sites our approach is able to outperform the competitive methods.

It would be interesting to design a method to efficiently compute the joint probability of multiple unpaired regions. Furthermore, the improvement of IntaRNA which get some benefit by considering seed features in comparison to RNAup, encourages us to take into account the existence of seed in the follow up work.

## Competing interests

The authors declare that they have no competing interests.

## Authors' contributions

RS participated in the design of the algorithm, performed the experiments, and drafted the manuscript. RB contributed to the design of the algorithm. SCS conceived of the study, contributed to the algorithm design, and supervised the project. All authors contributed to the writing of the manuscript.
